# A Bill to Promote the Science of Medicine and Surgery in the State of Illinois

**Published:** 1873-02

**Authors:** 


***A Bill to Promote the Science of Medicine and Surgery in the State of Illinois.***

SECTION 1. It shall be lawful in cities and counties whose population exceeds twenty thousand inhabitants, for superintendents of penitentiaries, wardens of poorhouses, coroners and city undertakers, to deliver to the professors and teachers in medical colleges and schools in this State, and for professors and teachers to receive, the remains or body of any deceased person, for purposes of medical and surgical study: *provided*, that said remains shall not have been regularly interred, and shall not have been desired for interment by any relative or friend of said deceased, within twenty-four hours after death: *provided, also*, that the remains of no person who may be known to have relatives or friends shall be so delivered or received without the consent of said relatives or friends: *and provided*, that the remains of no one detained for debt, or as a witness, or on suspicion of crime, or of any traveler, or of any person who shall have expressed a desire in his or her last sickness that his or her body may be interred, shall be delivered or received as aforesaid, but shall be buried in the usual manner: *and provided, also*, that in case the remains of any person so delivered or received shall be subsequently claimed by any surviving relative or friend, they shall be given up to said relative or friend for interment.

SEC. 2. And it shall be the duty of the said professors and teachers decently to bury in some public cemetery, the remains of all bodies after they shall have answered the purposes of study aforesaid; and for any neglect or violation of the provisions of this act, the party so neglecting shall forfeit and pay a penalty of not less than twenty-five nor more than fifty dollars, to be sued for by the health officers of said cities, or other places, for the benefit of their department.

SEC. 3. The remains or bodies of said persons as may be so received by the professors and teachers, as aforesaid, shall be used for the purposes of medical and surgical study alone, and in this State only; and whoever shall use such remains for any other purpose, or shall remove such remains beyond the limits of this State, or in any manner traffic in the same, shall be deemed guilty of a misdemeanor, and shall, on conviction, be imprisoned for a term not exceeding one year in a county jail.

SEC. 4. Every person who shall deliver up the remains of any deceased person in violation of, or contrary to, any or all of the provisions contained in the first section of this act, and every person who shall receive said remains, knowing the same to have been delivered contrary to any of the provisions of said section, shall each and every of them be deemed guilty of a misdemeanor, and shall, on conviction, be imprisoned for a term not exceeding two years in a county jail.

